# Towards atraumatic cochlear implant insertion monitoring using a hydraulic-based, cost-effective intracochlear pressure probe

**DOI:** 10.3389/fsurg.2025.1702151

**Published:** 2025-11-13

**Authors:** Walter Bernardi, Stefan Weder, Georgios Mantokoudis, Marco Caversaccio, Philipp Aebischer

**Affiliations:** 1 ARTORG Center for Biomedical Engineering Research, University of Bern, Bern, Switzerland; 2 Department of Otorhinolaryngology, Head and Neck Surgery, Inselspital, Bern University Hospital, Bern, Switzerland

**Keywords:** cochlear implant insertion, intracochlear pressure, minimally invasive surgery, real-time intraoperative feedback, surgical training platform

## Abstract

**Purpose:**

Cochlear implantation is an established treatment for severe sensorineural hearing loss, but residual preoperative hearing is often lost during the surgery, in part due to intracochlear pressure transients that damage cochlear hair cells. To enable real-time monitoring of insertion dynamics, we propose a cost-effective, remote pressure probe for continuous measurement of intracochlear pressure during cochlear implant surgery.

**Methods:**

The probe comprises a steel cannula placed at the round window, transmitting intracochlear pressure to a remotely positioned pressure sensor, thereby preserving surgical access.

**Results:**

We demonstrated effective pressure transmission across different cannula sizes (between 0.21 and 0.41 mm), validating the probe concept. In an artificial temporal bone model, sensor measurements during cochlear implant insertion showed a strong correlation with reference measurements of intracochlear pressure.

**Conclusion:**

We developed and validated a novel, cost-effective, hydraulic probe for atraumatic, real-time monitoring of intracochlear pressure during cochlear implant insertion via a round-window approach. Thereby, the proposed probe offers a feasible, quantitative, directly interpretable metric on cochlear implant insertion.

## 1 Introduction

Cochlear implants (CIs) are a well-established treatment for severe to profound sensorineural hearing loss (1). In recent years, inclusion criteria have expanded towards patients with functional preoperative hearing in the low frequencies, for whom preservation of intracochlear structures is essential. However, residual hearing is often lost during the surgery (2). This makes patients with good preoperative hearing, who could otherwise benefit from a CI, ineligible or hesitant to undergo the procedure. A key contributor to this is physical trauma to the cochlear sensory epithelia (3). Under normal acoustic stimulation, the delicate stereocilia of hair cells undergo displacements of less than 100 nm (4). Against this backdrop, it is no surprise that the manual placement of an implant in close proximity to these fragile structures carries a high risk of irreversible damage. Beyond immediate mechanical trauma, subtle and delayed neural damage has been reported in animal models (5). Limitations in kinematics of the human hand and events such as implant regrasping can cause strong intracochlear pressure transients, potentially severely impairing cochlear integrity (6, 7). Banakis et al. measured intracochlear pressure (ICoP) transients during CI insertions that correspond to sound pressure levels up to 174 dB SPL, which would permanently impair the patient’s residual hearing. The cochlea is completely encased by dense bone, which obstructs direct visual access and substantially limits the means to measure insertion-related processes. Within the insertion procedure, it is not possible to detect or quantify microtrauma to the microscopic sensory cells. Conventional imaging tools offer limited resolution and expose patients and operator to ionising radiation (8). Impedance measurements can provide structural and positional cues (9), and may indicate larger-scale trauma such as intracochlear bleeding (10). Electrocochleography can yield information about the cochlear integrity, but it is not reliably obtainable in all patients (11), and ambiguity in signal interpretation prevents its routine clinical application (12). Even combined, current tools are not sufficient to fully capture the dynamics of cochlear insertion.

Recent work has highlighted intracochlear pressure as a sensitive marker of insertion-related trauma, both in general trends across studies (13) and in specific events such as tip fold-over, which generate distinct high-amplitude transients (14). However, while intracochlear pressure is an established metric for cochlear stress in cadaver and in-vitro experiments, it has not been measured in clinical settings. Previous ICoP measurement studies utilised Fabry Perot (FP) pressure sensors or micromanufactured microphones (15). These solutions offer high resolution for measuring pressure in the confined sections of the *scala tympani* and *scala vestibuli*. In the work of Banakis et al., two FP sensors with a diameter of 260 μm were inserted via drilled cochleostomies (16), which makes this approach unavailable for clinical use. Furthermore, optical fibres require complex and expensive control schemes involving optical interrogators and the measurements are cross-sensitive to temperature.

A simpler, clinically compatible design was recently proposed by Kishimoto et al. in the context of neurosurgery. Their system uses a remote piezoelectric microelectromechanical system (MEMS) transducer coupled to a fluid-filled needle, enabling percutaneous pressure monitoring without direct electrical or optical components at the measurement site (17). Although promising, this approach has not yet been adapted or validated for intracochlear application.

In this work, we aim to contribute surgical training and cochlear implant research by providing real-time, quantitative feedback during electrode array insertion, using intracochlear pressure as a sensitive marker. To this end, we develop and validate a hydraulic sensor for real-time monitoring of intracochlear pressure during cochlear implant insertion. The sensor continuously tracks perilymph pressure transients, caused by the motion of the cochlear implant electrode array inside the *ST*. Its design centers around a fluid-filled cannula with a bent tip placed at the round window, transmitting pressure to a remote transducer. This sensor geometry preserves access for conventional electrode array insertion. By offering direct intraoperative feedback, the system aims to provide a novel quantitative metric to guide implantation, evaluate surgical tools, and assess the effects of implant design on insertion mechanics.

## 2 Materials and methods

In this paper, we design, validate and test a probe for monitoring intracochlear pressure transients via a remote, fluid-filled cannula. The probe is composed of a MEMS transducer, a cannula and an adapter connecting the two parts.

### 2.1 Pressure probe

The probe assembly consists of the following components:

• A syringe connected by a flexible tube for filling the cannula
• A commercial piezoelectric MEMS pressure transducer (MS5837-02BA, TE Connectivity) on a printed circuit board (PCB)
• A 3D printed housing with an anti-bubble adapter
• A luer-lock adapter for mounting of the cannula
• A sterile medical needle serving as the cannula

**Figure 1:**
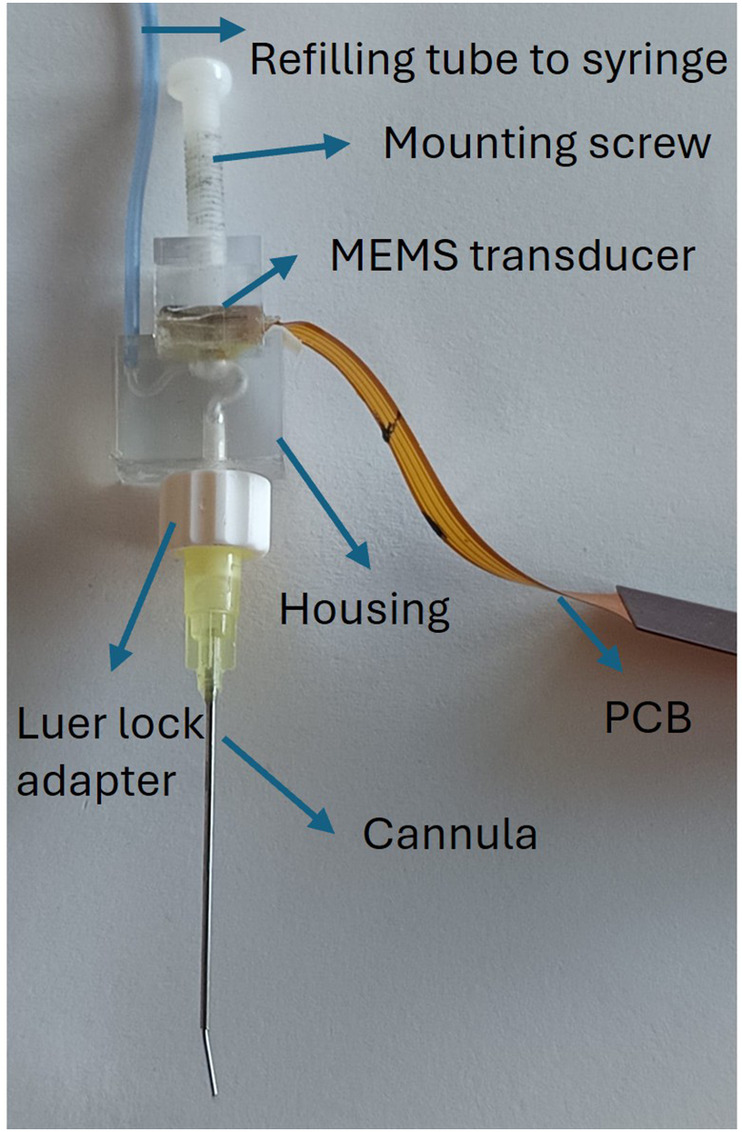
The probe assembly: the refilling tube connects a syringe to the housing inlet; the mounting screw holds the PCB-mounted MEMS transducer; the cannula is then connected to the housing outlet via a luer-lock adapter.

**Figure 2:**
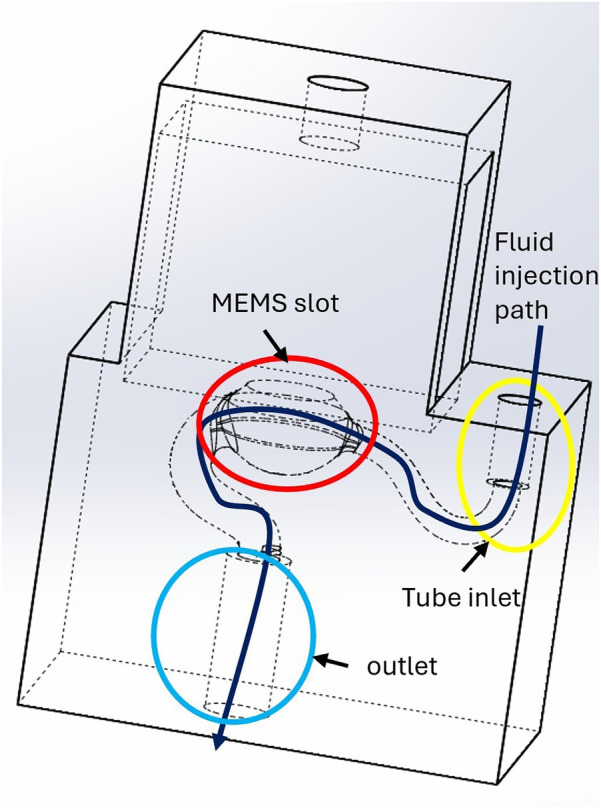
3D-printed sensor housing connecting the pressure sensor to the cannula. To ensure proper filling, physiological saline solution is flushed along a channel that runs across the housing. The red circle highlights the slot for the MEMS transducer membrane, the yellow circle indicates the tube inlet, and the blue circle marks the outlet. The dark blue arrow illustrates the fluid injection path.

As displayed in Figure 1, a MEMS transducer is mounted on a flexible PCB, which also hosts a protection circuit. The 3D printed housing is shown in Figure 2. For reliable pressure transmission from the cannula tip to the transducer, the cannula must be completely filled with physiological saline solution and air bubbles avoided. To address this, the housing features an anti-bubble adapter, composed of an S-shaped channel that runs past the pressure transducer. This channel allows a slow filling of the probe cavity therefore preventing the introduction of air bubbles. A screw pushes the pressure transducer against the sensor housing, creating a water tight seal. The housing is fabricated in a transparent material, which allows to visually verify correct filling.

Standard Luer-lock sterile needles are used as cannulas. To respect the anatomical constraint of the round window [long axis: 1.5–2.1 mm, short axis: 1.9 mm (18)], we selected three candidate cannulas with the following gauge level (Gs), outer diameter (ODs) and inner diameter (IDs):

• thin (27G: OD 0.41 mm, ID 0.21 mm; length 23 mm)
• medium (24G: OD 0.57 mm, ID 0.31 mm; length 23 mm)
• large (21G: OD 0.72 mm, ID 0.41 mm; length 23 mm)

For use in an anatomically correct model, we designed a stepped cannula that consist of the medium size needle at the tip, extended by a larger diameter base:

• base (20G: OD 0.91 mm, ID 0.60 mm; length 36 mm)
• tip (24G: OD 0.57 mm, ID 0.31 mm; length 6 mm)

The tip was manually bent with a forceps at about $16^{\circ }$, to accommodate the anatomy of the promontory and optimise visual access in a surgical situation. This stepped cannula is shown in Figure 1.

### 2.2 Electrical equivalent circuit

Electrical equivalent circuits provide an intuitive framework for describing and analysing the operating principles of physical systems.

For the proposed sensor, we can identify analogies between our physical variables and their equivalent circuit components:

• Pressure ∼ Voltage
• Fluid flow ∼ Current
• Fluidic resistance ∼ Resistance
• Fluidic compliance ∼ Capacitance

Besides the atmospheric pressure (direct current (DC) component), intracochlear pressure contains an alternate current (AC) component which are the pressure transients in which we are interested.

**Figure 3:**
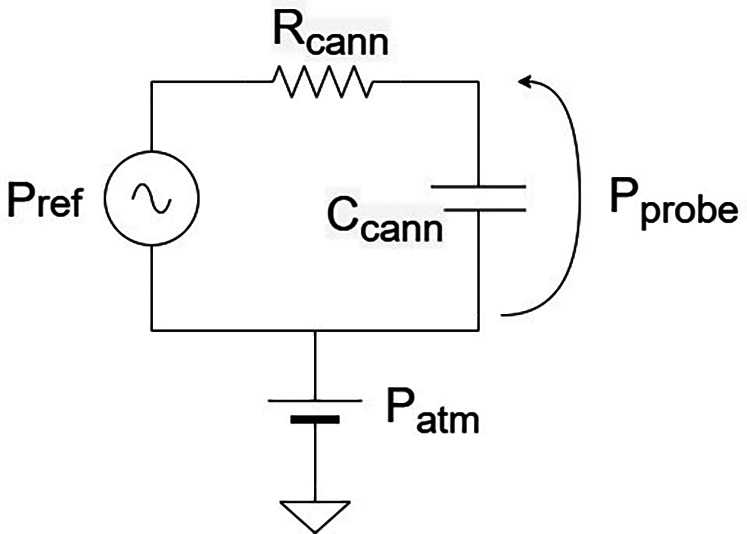
“Resistive–capacitive” circuit equivalent of the pressure probe.

The overall fluidic resistance depends on the cannula geometry, cochlear geometry and fluid viscosity. The dominant source of compliance arises from trapped air bubbles: because air is compressible, its presence attenuates transmitted pressure, reducing the effective bandwidth and smoothing transient signals. Figure 3 illustrates the corresponding electrical equivalent model.

The model captures the principal physical variables and accounts for the static and dynamic components of the pressure signal. In particular, beyond $P_{\mathrm{sens}}$ (the pressure measured by our probe) and $P_{\mathrm{in}}$ (the input pressure) we consider $P_{\mathrm{atm}}$, the atmospheric pressure level. As in a circuit, the output pressure (∼Voltage) can be determined by applying a “Voltage divider.”

$$P_{\mathrm{sens}}=\frac{Z_{C_{\mathrm{cann}}}}{Z_{C_{\mathrm{cann}}}+R_{\mathrm{cann}}}P_{\mathrm{in}}=\frac{1}{1+j\omega \tau_{\mathrm{cann}}}P_{\mathrm{in}}$$ (1)

where $R_{\mathrm{cann}}$ is the cannula hydraulic resistance, $C_{\mathrm{cann}}$ the cannula compliance, $Z_{C_{\mathrm{cann}}}=\frac{1}{j\omega C_{\mathrm{cann}}}$ and $\tau_{\mathrm{cann}}=R_{\mathrm{cann}}C_{\mathrm{cann}}$. In the static case, the capacitance behaves as an open circuit ($Z_{C_{\mathrm{cann}}}\to +\infty$, yielding $P_{\mathrm{sens}}=P_{\mathrm{in}}$, therefore the static pressure is fully transmitted.

In the dynamic case, the output pressure is transmitted without distortion only if the compliance of the fluidic circuit is negligible. The electrically equivalent model is an RC circuit, corresponding to a first-order Butterworth filter. As such, the system acts as a low-pass filter, with the cannula’s compliance and resistance jointly determining the sensor bandwidth. The hydraulic resistance depends on the cannula geometry describes the probe’s time constant $\tau_{\mathrm{cann}}$. According to Poiseuille’s Law, the hydraulic resistance of a straight cylinder scales as $\propto \frac{l}{r^{4}}$, where l is the cannula length and r is the radius. This highlights the dominant role of the radius, implying that the probe’s dynamic sensitivity is highly dependent on cannula diameter. Furthermore, compliance is strongly influenced by the presence of air bubbles, since their compressibility attenuates pressure transmission. These factors highlight the importance of selecting an appropriate cannula diameter and ensuring a clean filling process in order to achieve accurate, high-bandwidth intracochlear pressure measurements.

### 2.3 Probe validation

#### 2.3.1 Concept validation

In order to validate our sensor concept, we verified Stevin’s Law, which states that hydrostatic pressure scales linearly with height h:

P=ρgh (2)

**Figure 4:**
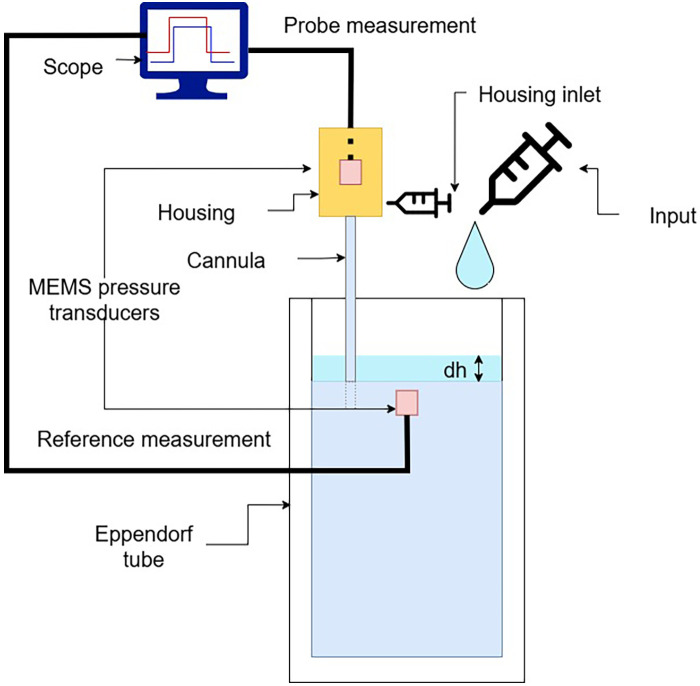
Validation setup used for assessing the probe response by measuring hydrostatic pressure at different water column heights in a test tube.

We compared our probe to a reference MEMS transducer fully immersed in a 50 ml sample tube. The setup is shown in Figure 4. The hydrostatic pressure was varied by adding and removing water in the sample tube manually using a syringe, creating stepped pressure changes. The procedure was repeated 10 times with three different cannulas. Between consecutive measurements, the cannula was refilled in order to observe repeatability of the filling procedure.

#### 2.3.2 Pressure dynamics

Referring to the equivalent circuit shown in Figure 3, the geometry of the cannula influences the dynamic transmission of pressure. According to Poiseuille’s law, the resistance of a cylindrical conduit scales with $\propto \frac{1}{r^{4}}$. Thus, the probe cannula radius must be carefully chosen, balancing damping of pressure transmission with anatomical constraints.

The equivalent circuit model is a linear, time-invariant system. Its response to a step input is an exponential rise, characterised by a time constant τ. To quantify this behaviour, we fitted the recorded pressure traces with a sum of exponential step functions:

$$P(t)=\sum_{i}P_{i}\left(1-e^{-\frac{t-t_{0i}}{\tau_{i}}}\right),$$ (3)

where P(t) is the measured pressure over time, $P_{i}$ is the amplitude of each pressure step, $t_{0i}$ the onset time, and $\tau_{i}$ the time constant of each step.

#### 2.3.3 Evaluation in temporal bone setup

**Figure 5:**
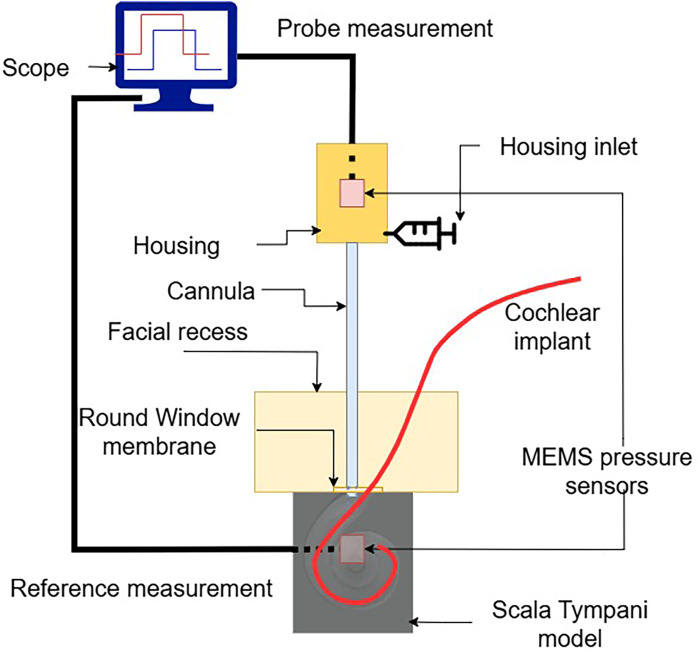
Test setup, to monitor ICoP during a real insertion.

To assess the sensor’s usability, we performed CI insertions in a previously validated, high-fidelity artificial model of the temporal bone. The model’s anatomy is based on human μCT scans and was extensively used in previous research (7, 19–22). To emulate the round window and retain perilymph perturbations within the cochlear model, a thin cross-slit sheet of parafilm was placed between the facial recess and the *ST*. The apex of the *ST* was connected to a piezoelectric pressure sensor, which we used as reference sensor, used in this configuration in (7, 21, 23). The facial recess and *ST* were securely anchored together. The fully mounted setup is shown in Figure 5.

The sensor tip was placed through the round window at the anterosuperior border, leaving adequate space for the insertion of the electrode array. The sensor was held in place using a flexible gooseneck arm with an alligator clip. In order to ensure the continuity between the water filling the cannula and the round window, a drop of physiological saline solution was further added around the sensor tip. A lateral wall CI electrode array (Flex28, MED-EL Elektromedizinische Geräte GmbH, Innsbruck, Austria) was inserted into the model. In particular, 12 insertions were performed by a trained engineer using surgical forceps.

#### 2.3.4 Statistical analysis

Pressure traces were retrieved synchronously from the reference sensor and our probe sensor. They were processed and analysed using the SciPy scientific computing library (24). To validate the sensor concept, we subtracted atmospheric pressure, calculated correlation and performed linear regression between the reference and our probe pressure recordings. We verified the system response shown in Equation 1, introducing the input pressure steps, which follow Equation 2. We fitted the model from Equation 3, using least squares, on the measurements to extract the time constants of the different cannulas. For the insertions performed *in vitro*, we performed the same analysis as the sensor concept validation.

## 3 Results

### 3.1 Probe validation

#### 3.1.1 Concept validation

**Figure 6:**
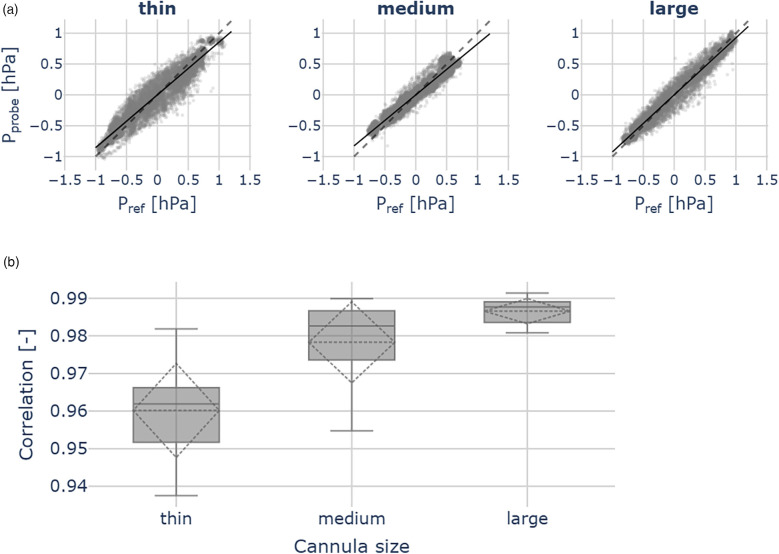
Probe validation outcomes across the thin (ID 0.21 mm), medium (ID 0.31 mm) and large (ID 0.41 mm) cannulas, for each cannula 10 measurements were performed. **(a)** Scatter plots show pressures measured by the reference sensor ($P_{\mathrm{ref}}$) and the hydraulic probe ($P_{\mathrm{probe}}$). The dashed line indicates identity. **(b)** Correlation between hydraulic probe and reference sensor pressures. Across all cannula sizes, we observed good agreement with the reference measurements.

For each cannula size, we observed good agreement with the reference measurements. The median Pearson correlations for each group are: 0.96 (thin cannula), 0.98 (medium), 0.99 (large) (Figure 6a).

Linear regression yielded the following slope coefficients [95% confidence level (CL)]:

• thin cannula: 0.86 ([0.82, 0.90]), $R^{2}$ = 0.92
• medium cannula: 0.83 ([0.79, 0.87]), $R^{2}$ = 0.96
• large cannula: 0.92 ([0.90, 0.94]), $R^{2}$ = 0.97

We further observed the good correlations between the remote probe and the reference pressure measurements across all cannula sizes. Although the correlation coefficients differ significantly (ANOVA: F = 17.34, *p* < 0.05), they remain close to 1.00, indicating robust agreement between the reference and the remote probe (Figure 6b).

#### 3.1.2 Pressure dynamics and cannula choice

**Figure 7:**
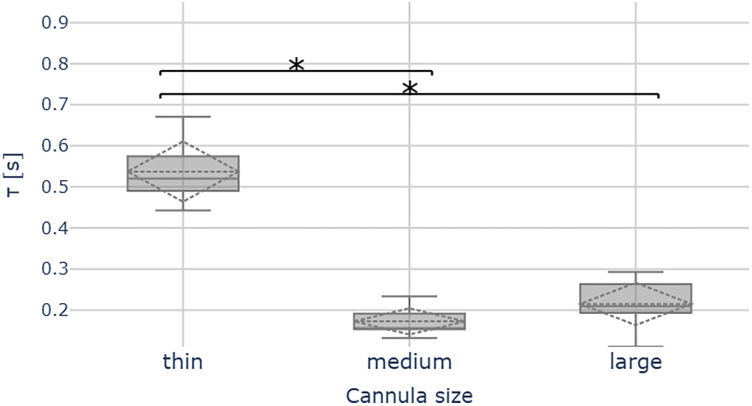
Time constants extracted from pressure measurement traces. The medium-sized cannula (OD 0.6 mm) represents a good compromise between the sensor bandwidth and surgical access.

In Figure 7 we show the time constants extracted from pressure traces for the three separate cannulas. For each cannula, we computed the time constant of 9 subsequent pressure steps, present in a single trace, by fitting the sum of exponential responses, according to Equation 3. Time constants τ across the groups present significant differences (ANOVA: F = 93.61, *p* < 0.05). In particular, the strongest difference is given by the thin cannula reaching a median τ= 0.52 s, approximately 2.5 times larger than the other two (τ = 0.2 s). Between the medium and large cannula, no significant difference was observed.

#### 3.1.3 Evaluation in temporal bone setup

**Figure 8:**
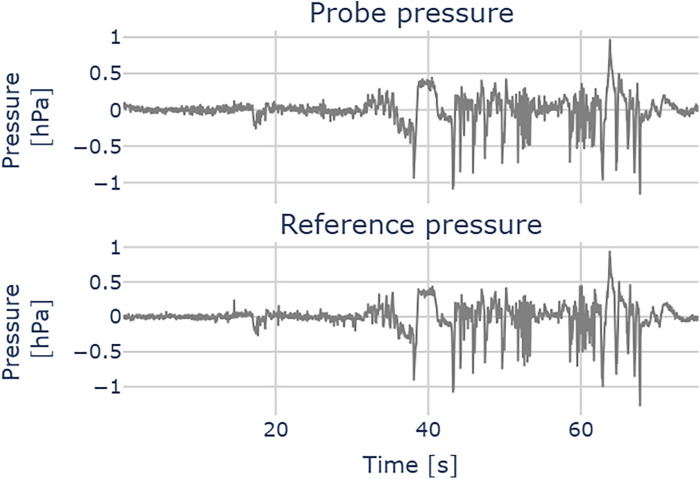
Pressure measurement during a cochlear implant insertion. Upper plot: probe pressure; lower plot: reference pressure.

**Figure 9:**
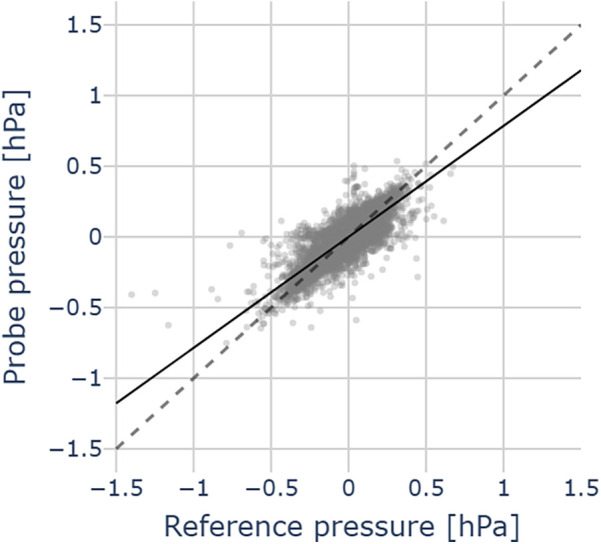
Pressure comparison between probe and reference pressures in 12 CI insertions in an artificial temporal bone model. Data points (grey dots), mean regression slope (black line), the grey dashed line represents the identity between probe and reference pressures.

Figure 8 illustrates a pressure measurement during cochlear implant insertion. The computed residuals between the two traces show a mean of 0.00 hPa and a standard deviation of 0.05 hPa, suggesting no systematic bias. The standard deviation is in the order of 2x root mean square (RMS) resolution of the pressure transducer. We successfully used the probe across all the 12 CI insertions. Figure 9 shows the relation between our probe and the reference measurement. The mean slope of the fitted linear regressions is 0.79 (95% CL [0.73, 0.84], mean $R^{2}$ = 0.66).

## 4 Discussion

In this study, we introduced and validated a novel probe concept for real-time monitoring of intracochlear pressure during cochlear implant insertion. The probe is designed to preserve surgical access and was evaluated in a high-fidelity artificial temporal bone model, providing a realistic *in vitro* setting to assess its usability and performance.

### 4.1 Cannula diameter

The equivalent circuital model of the presented probe provides an intuitive framework for understanding the probe’s operating principle. The dynamic response depends on both fluidic compliance and resistance of the system, which determine the sensor bandwidth. The compliance is primarily influenced by air bubbles, and can be minimised by slow filling and visual inspection. Hydraulic resistance, which scales according to $\propto \frac{1}{r^{4}}$, further limits bandwidth as the cannula radius decreases. For surgical use, the cannula diameter should be as small as possible to preserve access, but excessive miniaturisation reduces sensitivity to rapid pressure transients. Our characterisation of three cannula sizes showed that the medium-sized cannula (ID 0.31 mm) provides the best compromise, offering sufficient dynamic sensitivity while remaining surgically practical.

### 4.2 Probe validation

#### 4.2.1 Concept validation

As shown in Figures 6a, 6b, the hydraulic pressure is effectively transmitted to the piezoelectric MEMS transducer embedded in the housing via the water column within the probe cannula. Imperfect matching is likely attributable to sensor noise, which affects the regression results.

#### 4.2.2 Pressure dynamics

The time constants extracted for the probes, as shown in Figure 7, can be seen as confirmation of the pressure transmission in a dynamic regime, and characterise the probe speed. The response of the thinnest cannula is too slow for practical purposes (τ=0.52 s), as it would be insufficient to track sharp pressure transients, indicative of traumatic events during insertion (7).

The medium and large cannula diameters show similar time constants with differences on the order of the sampling interval ($t_{\mathrm{sampling}}=25$ ms), which is negligible. Considering that they do not show a significant difference in their dynamic response, we suggest using the medium (OD 0.57 mm) cannula, as its smaller footprint improves placement and visual access within a surgical setup.

#### 4.2.3 Feasibility in intraoperative context

We successfully reproduced pressure transmission across the cannula also during CI insertions in an artificial temporal bone model (see Figure 9). This confirms the usability of our sensor for intracochlear pressure monitoring. Compared with the concept validation experiments (Figure 6b), the agreement with the reference sensor (placed in the apex) is slightly reduced in this simulated intraoperative context. One possible explanation for the pressure mismatch is that the reference sensor itself is subject to a dampened pressure signal, compared to the pressure signal caused at the base by the implant insertion. We suspect that the presence of air bubbles may increase compliance in the cannula and dampen the pressure transients. The movement of the implant during the insertion of the CI may be a cause of bubble formation. These may surround the cannula tip or even enter it, therefore attenuating the pressure readout. This effect could not be completely suppressed, but it did not compromise the sensor’s ability to detect pressure transients. Surgical feedback on probe usability during cadaveric CI insertion would be valuable, in order to assess the feasibility of a trans-round window membrane (RWM) approach.

### 4.3 Probe placement

**Figure 10:**
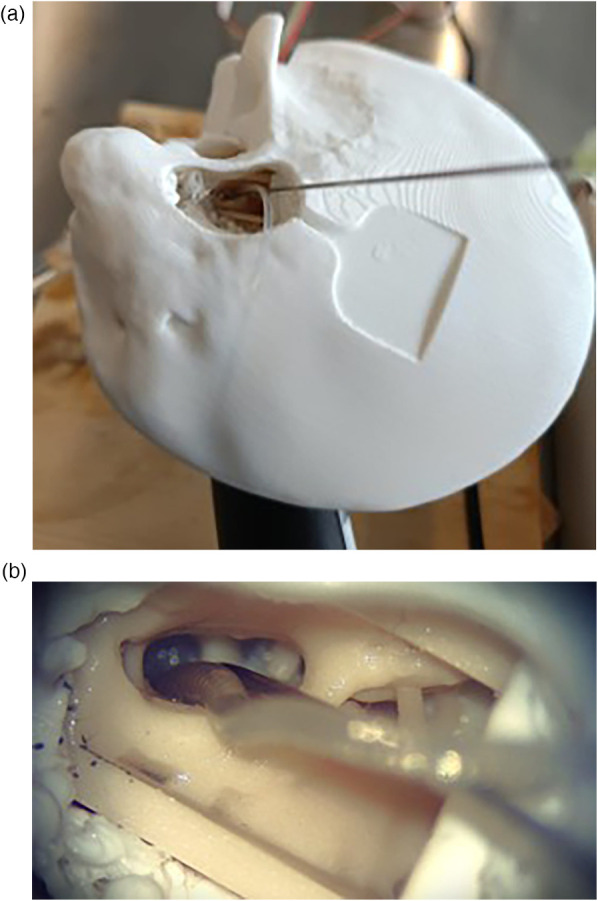
Views of the sensor cannula and CI placement from the surgical microscope. **(a)** Full temporal bone. **(b)** Facial recess.

The rationale for a round window measurement approach is to avoid creating an additional cochleostomy to enable clinical application (16, 25). The round window measures approximately 1.5 mm by 2 mm, and can therefore accommodate a cannula diameter of up to about 1 mm alongside the cochlear implant. This makes the round window application of the probe feasible. The cannula placement is shown in Figure 10. A key advantage of the proposed design is that cannula curvature does not impair pressure transmission, allowing the probe geometry to be tailored for optimal access to the ST through the complex anatomy of the facial recess and round window niche.

#### 4.3.1 Impact of round window opening

If the tip is placed through the RWM, but the cochlear opening is large and in communication with the promontory, transients induced by the CI movements may be attenuated to amplitudes approaching or even below the transducer resolution (1–10 Pa). In insertion studies within an *in vitro* model, Todt could show that a punctured artificial RWM resulted in a significantly larger pressure compared to half-open and fully-opened RWM (23). We can interpret this outcome by referring to the circuit in Figure 3, in particular, adding a compliance element in parallel with the sensor compliance. This additional compliance can reduce the bandwidth of the probe. As a result, the half-open and fully open RWM configurations lead to transient dampening.

#### 4.3.2 Underwater pressure measurement

Filling of the middle ear with physiological saline is an established method in cochlear implantation known as the underwater technique (26). In this configuration, the intracochlear volume remains in continuous contact with the extracochlear fluid, theoretically allowing the possibility of measuring pressure outside the cochlea. However, in the case of an extracochlear probe placement, the pressure input (produced by the CI movement) would face additional resistance imposed by the geometric constraints of the round window and a large parallel compliance. The consequence of this is the stabilisation of the pressure, which is coherent with (26). Although this may hinder the detection of rapid ICoP transients, pressure equalisation could help protect the intracochlear structures, supporting atraumatic CI insertion. Future finite element simulations of the fluid dynamics during wide opening of round window (RW) and underwater during CI insertion could provide deeper insights into the transient behaviour, anatomy-related effects, and probe performance.

### 4.4 Manufacturing

The design of the pressure probe is oriented towards a rapid prototyping approach. Moreover, compared to the probe by Kishimoto et al. (17), we avoid silicone oil, which could pose cytotoxic risks (27). In our pressure probe, despite careful fluid injection to maintain the fluid-filled column, it is unlikely to completely eject air bubbles trapped inside the cannula, which could attenuate the probe response. Since the cannula is a non-transparent steel needle, we could not exactly verify the presence of bubbles inside it. For clinical deployment of the remote pressure probe, we identified the following manufacturing improvements:

• Optimised cannula curvature and length, to free the surgical view, hence placing the housing and electronics aside. With a good filling, we expect the extended length not to impact the measurement output, as the length contributes only as a linear factor to fluidic resistance.
• Conformal membrane coating; as surgical counterparts require probe hermeticity, we suggest a coating as performed in (17), for example via physical vapor deposition (PVD).
• Cannula with a tapered tip diameter at the round window.
• More compact cannula-to-transducer adapter.
• High resolution pressure transducer, to improve detection of attenuated pressure transients.
• Sterile-packaged base sensor.

The current probe assembly cost is estimated at around $15, making it substantially more affordable than comparable pressure monitoring setups. Adopting precision manufacturing procedures may increase costs, mostly due to the processes (i.e., membrane coating). However, this would be leveraged by the compelling advantage of easier and facilitated MEMS readout.

## 5 Conclusion

We developed and validated a cost-effective hydraulic probe for real-time intracochlear pressure monitoring during cochlear implant insertion. The design is based on a fluid-filled cannula, positioned at the round window, that transmits pressure to a remote MEMS pressure transducer. It allows atraumatic placement and preserves surgical access by enabling flexible choice of the cannula geometry. Concept validation across cannula sizes confirmed accurate static pressure transmission and showed that even cannula diameters below 0.60 mm maintained rapid dynamic response. In a high-fidelity artificial temporal bone model, the probe reliably captured insertion-induced pressure transients with close agreement to an intracochlear reference sensor. These results demonstrate the feasibility of hydraulic coupling for intracochlear pressure sensing. Our tool can enhance surgical training by providing direct feedback on insertion dynamics, and enable quantitative comparison of new electrode designs and surgical instruments. While this study was conducted in vitro, future work will focus on ergonomic refinements, developing sterile, clinically compliant versions of the probe, and validating the system in cadaveric and clinical settings. Integration with existing surgical workflows could ultimately provide surgeons with a quantitative intraoperative metric to reduce insertion trauma and improve hearing preservation outcomes.

## Data Availability

The raw data supporting the conclusions of this article will be made available by the authors, without undue reservation.
